# Supplementary material for: Identification of Iguania Ancestral Syntenic Blocks and Putative Sex Chromosomes in the Veiled Chameleon (Chamaeleo calyptratus, Chamaeleonidae, Iguania)
Source: Int J Mol Sci. 2022 Dec 13;23(24):15838. doi: 10.3390/ijms232415838 (PMC9779593; doi:10.3390/ijms232415838)
Supplement: Supplementary file 1 [file ijms-23-15838-s001.zip › Supplementary_materials.v.3.pdf]

Table S1. Primers for PCR of *Chamaeleo calyptratus* repetitive DNA monomers.

Name	Sequence (5′-3′)	Length, bp	T_m °C
CCA_s1_F	CAAACCACATATGAGGCAATAC	22	60
CCA_s1_R	ACCCTCACAAACTACACTATG	21	60
CCA_s2_F	GACAGCCCTGTTCATTCT	18	60
CCA_s2_R	GGCTGTCAAGAATCCCTTAT	20	60
CCA_s3_F	TGCTTCACTGAATGGGATAG	20	60
CCA_s3_R	GTGGGAGTGTCCTTGTAATAG	21	60
CCA_s4_F	CCAGCTTCTTCCACCAC	17	60
CCA_s4_R	GAAGCAAGCATGGGAGAA	18	60
CCA_s5_F	CCAACTCTGGGTCCATTT	18	60
CCA_s5_R	CCCTAACCCTAACCATAACC	20	60

Table S2. Monomeric consensus sequencing of identified *Chamaeleo calyptratus* repetitive DNAs. Primer sequences are underlined.

Repetitive element	Sequence with underlined primers sites
CCA_s1	AGCAAACCACATATGAGGCAATACACAGTTCCCTTATGGATGTTGAG<br>TTTGTTCCAAATGAGAATTCAGTACAAATATAGATCAAAAGATTTCTG<br>TTGTTCTCCTAATATGTTATACCAATACATTTGGTATATCAAATAGAA<br>GATGAGGAACATGTTTGCTAGAACTCCCTCGGTCAAAACTAACAAGG<br>CCAAATTGATTGCACCTGTTGGTTAGTTCATTCTGCAGTTCCATTTCC<br>CTATCATAATGTGACAAAGATTAGATGCCAACATAAGTTTGCTATTA<br>CAAGGGTACACCGAATTGTGATTAACTGAATCAGAATGGGTTATTCT<br>GGAATAGCAATTCCTATGTCTGCATTGGAGATGTACATAATTCCAAG<br>ATACATTTGAAACACTGAAGCTAAAATGATGTCCCGTAACATAGTTC<br>TCTTTCCTGAAGTTATCCATAGTGTAGTTTGTGAGGGTTT
CCA_s2	ATTCTTGACAGCCCTGTTCATTCTGAGCTCTCTGCTGCCTTCCTGCATA<br>TCTATATTTAGATTTTAAAAATCATGGGAAGTTTCCCTGAAAATAAGG<br>GATTCTTGACAGCCCTGTTCATTCTGAGCTCTCTGCTGCCTTCCTGCAT<br>ATCTATATTTAGATTTTAAAAATCATGGGAAGTTTCCCTGAAAATAAG<br>GG

CCA_s3	TACTTTTATTCTGACCAAGTGTGAAAAGATTAGTAGGAGCCACAACG<br>TTGCTATTTCCATTCACTGAACCGGATTTTGCTTCACTGAATGGGATA<br>GCGCTGTGCGGTGTACTAGAACTTCTTTTCTTTACACTACAGTTATAG<br>GGAATATCCGTATACATCCAAAACACTGCAACAAACAATGTACCATA<br>TCAAAGTCGACTTTGCTACAGCTACCCATCGTGGTGCTAGTTGTAGTA<br>TAGGCAAACCACAATGGAGGTCATAAACATGTGCAATATTGTATAGT<br>ATTTTGCTCTGCTTAGAAATGAAACACAAGTAGTGAATGAGGTTTTC<br>CTCTTGTTCAGCTGGTTTGACGCACTACCACATTTGCCATTTCACAC<br>AGGAGAGAAAGAACATGTTTGCTAGAATCTCCTTCGCCTAATATGA<br>ATGATGCAAAACGATGGCACGTGTGGGTGACTTTATTTAGCAGTTC<br>CTTTGACCAAGTGTGAGAAAGATTAGTTGCCAATGCAAGTTTGCTA<br>TTACAAGGACACTCCCACTTGTGGTTCAGCGAATGGGAAAGTGCTA<br>TATTATGTACTGACATTCTTATGGACACAGTGTAGTTATTGGGAATT<br>CCATTATACATTAAAAACCCTGAAACAAACTTAATGTCAGATATCA<br>TAGTTGTTTTGGCTATAGATACCCATAGTGCATCTTGTAGATGAGTG<br>GGCACTCTTCATAGGAGCTTATTCGTATTCCATAAGCCAGAAAGGA<br>ATCATGGAAAACGATGGAACCTCTTTGTGACTTCATTCTGCCGTTCA<br>TTTTCCTTCACCTGGAGTGACAAAGGCTAGAGAGCCAGGCAACATT<br>GTTATTAACAAGGTAAAAACAATTGTGGTGCATTGAATGGGAAACG<br>GCTGTATTATGTAATAGGAATTGTTATATCTACACTACAGTTATGGG<br>GAATATCAGGACACATCCAAAACACTGCAACAATCTTAGTGTCCCA<br>TATCATAGTCGTCTTGCCTAAAGCTACCCATAGTGGAGCTAGTTGAA<br>GTATTGGCAAACCACATATAACGTGATATACAAGTCCATTATTATTT<br>AATAATTTGGTCTACATATGAACGCGGCTGATATGTCAGATAACCA<br>TATTTGTCATTTCACACAGGAGAGGAGGAACATGTTTGCTAAAATG<br>CTCTGCAGCAGAACTGAATCTTGTAAAGGTGTGGCAAATCTTGGTG<br>ACTTTATTGTGCTTT
CCA_s4	TTCTGCCAGCTTCTTCCACCACCCCTTCTCCCATGCTTGCTTCCCCCATT<br>T
CCA_s5	CTCCTACCTGGAGAATACATGAACGATTCCTCGCGCATGCTACTTGTTA<br>CCTAGTGCCCGCTGGGAAATAAGGTTATATTTCATTTCTACTGTTTTA<br>GTATTTATATTTAAATTGTATTTTGTATTTTTCATTGTAAATCGCCCTGA<br>ATCACATTTTTCTTGAGTAGAGCTGGGTATTAATCTGATAAAACTAACG<br>GAATCAAATCGAGGTTTAAGGTTAGGGATAGGTTTTGGTTTAGGGTTCG<br>GGTTTGTCTTAAGATAGGAACAAAGGGTCAGATTCCTGAGCCATCTGCT

	TTCTAGCGACTCTTTCTTGCTGCCTAGTGTCTGCTAGAAATAGTATATCG<br>CTTTCTGTGTCGTATTTTGAACTAAGTGGCGGGTGTCGGATGCCGGGTG<br>CCGGGTGCCGGGTGCCACGTCCAACTCTGGGTCCATTTTCAACTCTGGG<br>TAGCGAATTCCGTTTGGCAAAGTGCCATTGGACAAACATGGCTGACCA<br>AGGGGGGCAGGCCAAGGGGGGGTGACCAAAGTCCGAGGGCCGGGGGC<br>CGGTGCTTTAAGTCCGTTGACAATTACTCATTGGGAAGGTGATTTTGGT<br>CAGATATCAAAGGCTAGATGCCACGGGTATGAAAAGACCGTCTGGGTG<br>ACACCATCCTGCTGATGTGCCCAAGGGAGGGTTGCCAAGTGGGGGGTG<br>TCCAAAGTCTGGGGGCCGGGGGCCGGGGCAAGGGCCGAGGGCCGAAG<br>ACCAGGGATCAAAGCCTGAGTGTTGAGGGACATTCCTAATCATGAAG<br>GTGCATTTCACCATTCGCAAGACATGGCAAGTGTTGTTATGGTTATGG<br>TTATGGTTAGGGTTAGGGTTAGGGTTAGTGAGCGACGCGGGAGACGCC<br>TGCAAGT

Table S3. Statistic of sequencing and mapping of flow sorted chromosome-specific DNA pools of *Chamaeleo calyptratus*.

Chromosome-specific DNA pool	# Reads	Reads, bp	# Target ACA	# Target PPL	# Target STR
CCA1	345354	83188216	49705	51839	53052
CCA2	304152	70953446	39872	42158	43049
CCA3	259350	62490046	33877	38762	39944
CCA4	250708	60979524	35256	40127	40524
CCA5	290126	69105853	37688	47464	44317
CCA6	289408	70864623	42473	49634	51478
CCA7	305316	75951315	41085	49699	62780
CCA8	301578	73654036	44130	47085	56034
CCA9	243536	61066427	39167	45936	46459
CCA10,11	238190	60845743	31625	39263	40912
CCA12	307186	75372625	34318	41158	44862

Reads - Reads bp the initial number and volume of sequence reads,

Target ACA - the number of reads mapped to AnoCar2.0 genome with MAPQ >20 after human contamination removal;

Target PPL - the number of reads mapped to MUOH_PhPlat_1.1 genome with MAPQ >20 after human contamination removal;

Target STR - the number of reads mapped to ASM1680106v1 genome with MAPQ >20 after human contamination removal;

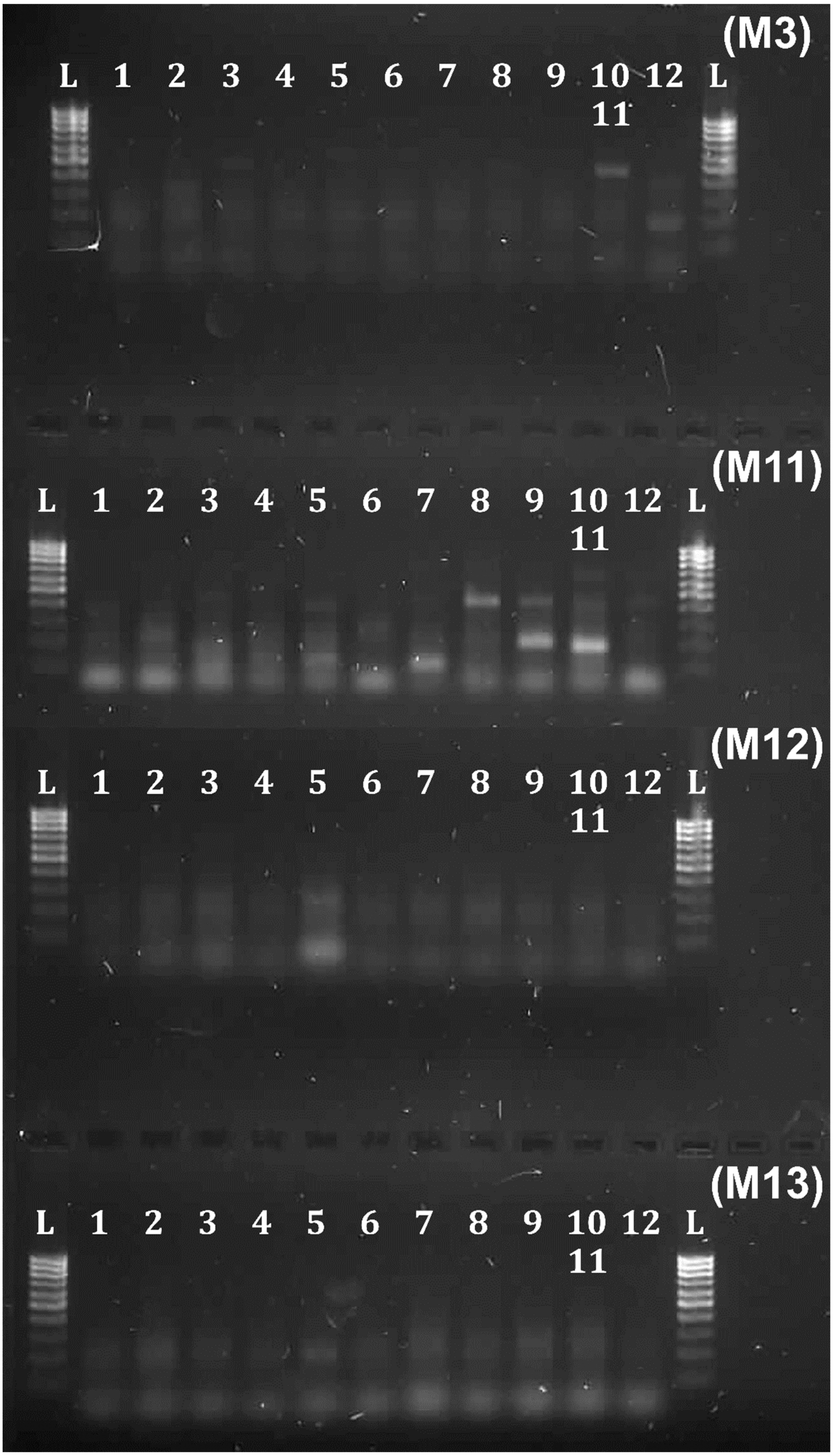

Figure S1. Agarose gel electrophoresis showing the result of PCR-assisted mapping of the male-specific RAD-seq markers in *C. calyptratus*. Numerals refer to the chromosomes, which were identified in the chromosome-specific DNA libraries prepared from *C. calyptratus* flow-sorted chromosomes, L DNA ladder.

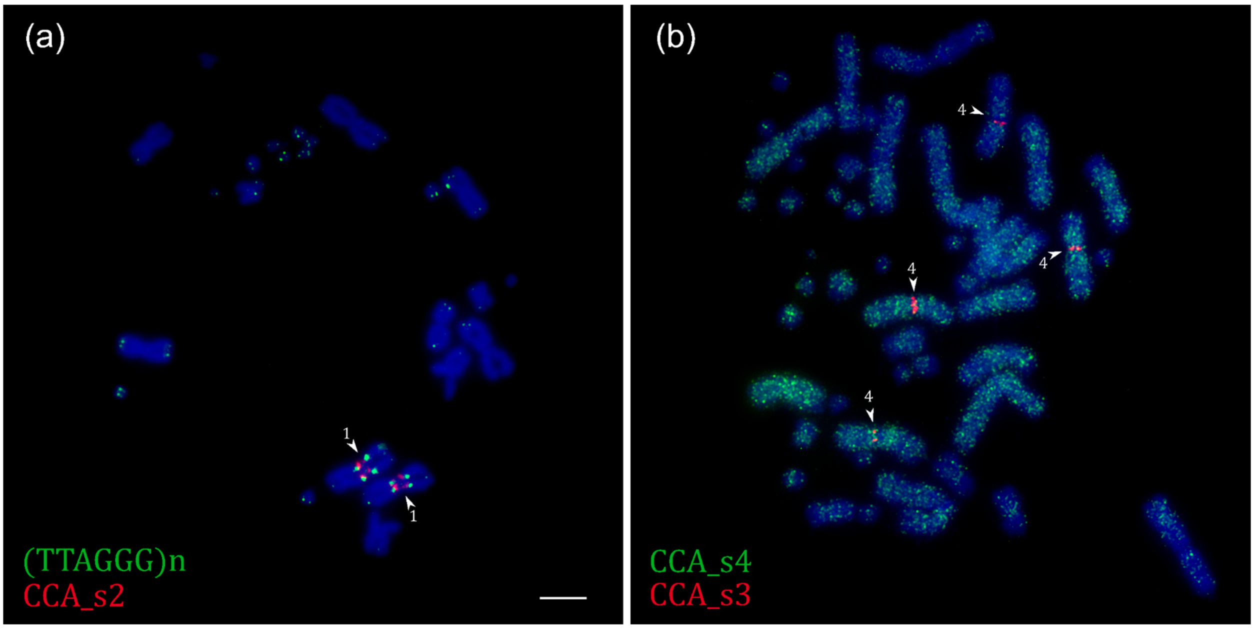

Figure S2. Fluorescent *in situ* hybridization of *C. calyptratus* repetitive element probes on its metaphase plates. Chromosomes are indicated by the arrow-heads. Colocalization of probes are shown: (a) (TTAGGG)n (green) and CCA_s2 (red); (b) CCA_s4 (green) and CCA_s3 (red), this metaphase plate is 4n (48 chromosomes). Scale bar = 10 um.

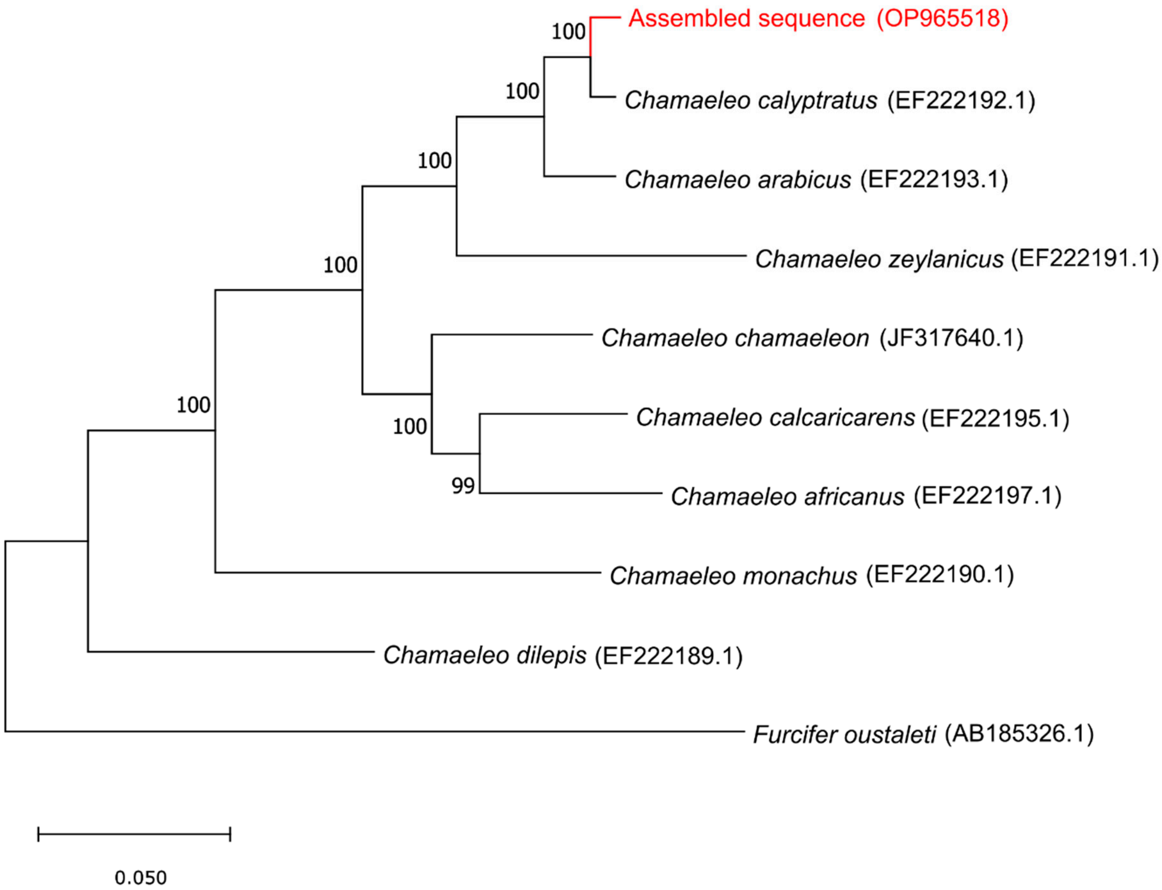

Figure S3. Phylogenetic relationship between the sample of *C. calyptratus* (red color on the tree) and *Chamaeleo sp*. The phylogenetic tree was constructed using available complete mitochondrial

DNA sequences from the NCBI Nucleotide database by the maximum likelihood method (1000 iterations) in the MEGA 11 software suite. The *Furcifer oustaleti* was used as an outgroup.
